# The Efficacy of* Brucea javanica* Oil Emulsion Injection as Adjunctive Therapy for Advanced Non-Small-Cell Lung Cancer: A Meta-Analysis

**DOI:** 10.1155/2016/5928562

**Published:** 2016-12-06

**Authors:** Wei Xu, Xinchan Jiang, Zhengyuan Xu, Tong Ye, Qionghua Shi

**Affiliations:** School of International Pharmaceutical Business, China Pharmaceutical University, Nanjing, Jiangsu 211198, China

## Abstract

*Purpose. *To evaluate the efficacy of* Brucea javanica *oil emulsion injection (BJOEI) in patients with advanced non-small-cell lung cancer (NSCLC) during chemotherapy.* Method. *Electronic database of EMBASE and PubMed and the conference proceeding of ASCO, CNKI, CBMdisc, VIP, and Wanfang database were searched to select RCTs comparing BJOEI plus chemotherapy with chemotherapy alone in the treatment of advanced NSCLC, until June 1, 2016. Two reviewers independently performed the analysis according to the inclusion and exclusion criteria. Review Manager 5.3 and STATA 12.0 were employed for data analysis.* Result. *Twenty-one studies including 2234 cases were included. The pooled result indicated that there were significant differences in ORR (RR = 1.25; 95% CI: 1.14–1.36; *P* < 0.00001), improvement of QOL (RR = 1.87; 95% CI: 1.63–2.15; *P* < 0.00001), nausea and vomiting (RR = 0.67; 95% CI: 0.46–0.98; *P* = 0.04), leukopenia (RR = 0.63; 95% CI: 0.52–0.75; *P* < 0.00001), but there was no difference in thrombocytopenia (RR = 0.78; 95% CI: 0.49–1.23; *P* = 0.29). Begg's funnel plot and Egger's test indicated that no publication bias was found. The sensitivity analysis suggested the stability of the pooled result.* Conclusion. *The addition of BJOEI can enhance efficacy, improve QOL, and decrease incidence of nausea and vomiting and leukopenia for advanced NSCLC patients. However, higher quality RCTs are needed to further confirm this finding.

## 1. Introduction

As a prevalent and highly malignant carcinoma, lung cancer is the leading cause and the second leading cause of cancer death among men and women worldwide, respectively [1]. While the incidence of lung cancer has declined in some regions like North America and Europe, it is still high in China with the incidence rate of 733.3 per 100,000 in 2015 [2]. Moreover, with an annual growth rate of 26.9%, the expected number of cases of lung cancer will reach 100 million in 2025, indicating that China might have the largest population of lung carcinoma patients around the world [3].

Lung cancer can be divided into two types: one is small-cell lung cancer and the other is non-small-cell lung cancer. It is estimated that the latter, of which the most common types are squamous cell carcinoma, large-cell carcinoma, and adenocarcinoma, accounts for 80%~85% of all global lung cancer cases [4]. The treatments of non-small-cell lung cancer (NSCLC) give first place to surgery, but most of the patients cannot receive the appropriate resection as they have reached an advanced stage when being diagnosed [5]. Standard first-line treatment for advanced NSCLC involves a combination of two drugs, including a platinum compound and a nonplatinum compound, such as paclitaxel and docetaxel, having indeed achieved favorable outcome [6]. However, the substantial toxicity incurred by chemotherapy, including gastroenteric reaction, hematotoxicity, nausea, and vomiting, should not be overlook, which is negatively correlated with quality of life (QOL) for NSCLC patients [7], especially for the advanced ones. Thus, how to reduce the burden of toxicity and achieve higher quality of life is the top priority on the clinical research agenda [8].

Traditional Chinese Medicines (TCMs) have become increasingly popular in the treatment of cancer in China [9].* Brucea javanica *oil emulsion injection (BJOEI) is one of TCMs products, which takes Brucea Jen petroleum ether extracts as raw material and purified soybean lecithin as emulsifier [10], and is employed as adjunctive therapy in the treatment of lung carcinoma, brain metastasis of lung carcinoma, and gastrointestinal tumorigenesis. A great number of published studies have proved that BJOEI can perform a synergetic antitumor effect by improving tumor response, boosting Karnofsky Performance Score (KPS), reducing the incidence of adverse events and stimulating the immunity during chemotherapy or radiotherapy [11]. The pooled result of a meta-analysis showed that the addition of BJOEI to chemotherapy produced favorable outcomes for patients with advanced gastric cancer, including improvement of objective response and QOL and reduction of side effects such as neutropenia, thrombocytopenia, nausea, and vomiting [12].

BJOEI has been applied in clinical practice for advanced NSCLC patients since long time ago, but no relevant meta-analysis was conducted. Thus, we perform this meta-analysis to investigate the clinical efficacy of BJOEI plus chemotherapy in the treatment of advanced NSCLC.

## 2. Methods

### 2.1. Literature Source and Search Strategy

We searched and extracted eligible studies about BJOEI treatment of NSCLC from databases of PubMed, EMBASE, the conference proceeding of American Society of Clinical Oncology (ASCO), Chinese Biological Medical disc (CBMdisc), Chinese National Knowledge Infrastructure (CNKI), Chinese Scientific Journals Full-text Database (VIP), and Wanfang database. The key words applied in the search were as followed: “lung cancer”, “non-small-cell lung cancer”, “NSCLC”, “*Brucea javanica *oil emulsion”, “BJOEI”, “Yadanzi”, and “chemotherapy”. The retrieved studies were regarded as potential source and reviewed manually. Moreover, although the published year of these literatures were unlimited, only English and Chinese literatures were accepted.

### 2.2. Inclusion and Exclusion Criteria

The following criteria were used for the literature inclusion. (1) The study design was confined to randomized controlled trials (RCTs) comparing platinum-contained chemotherapy alone with platinum-contained chemotherapy plus BJOEI for the NSCLC. (2) Study subjects who (a) were patients with stage III or IV NSCLC diagnosed pathologically and (or) cytologically; (b) had KPS ≥ 60 and (or) time of survival ≥ 3 months; (c) had outcomes of objective response rate (ORR) determined by World Health Organization (WHO) criteria or Response Evaluation Criteria in Solid Tumors (RECST), improvement of QOL evaluated by KPS, and adverse reactions assessed by WHO Recommendations for Grading of Acute and Subacute Toxicity; (d) had no chemotherapy contraindication before treatment and no significant abnormalities in liver, kidney, and heart function. The major exclusion criteria were as follows: (a) non-RCTs studies; (b) animal experiments, review, and other irrelevant studies; (c) no detailed data about ORR, improvement of QOL, and adverse events or no indicators for them; (d) single-arm study.

### 2.3. Endpoint Indicator

The outcomes included clinical efficacy, quality of life, and adverse effects. According to WHO criteria and RECST, the tumor response included complete response (CR), partial response (PR), stable disease (SD), and progressive disease (PD). The ORR was defined as CR + PR. Toxicity was graded from 0 to IV in severity on the basis of the WHO Recommendations. This meta-analysis only investigated the incidence of Grade II or above nausea and vomiting, leukopenia, and thrombocytopenia.

### 2.4. Data Extraction and Quality Assessment

Two reviewers independently extracted the information of the included study including name of author(s), publication year, number of patients in BJOEI group and control group, age, sex, chemotherapy regimen, stage of cancer, initial KPS, method of outcome ascertainment, study outcome, and detail of BJOEI treatment. Disagreement and problems were resolved by discussing or consulting with another reviewer according to the Cochrane handbook. The general methodological quality of each included trials was assessed by six items according to the Cochrane Collaboration's Risk of Bias (ROB) criteria. The items are about randomization, allocation concealment, double blinding, integrity of outcome data, selective reporting, and other bias. Every item is given a possible score of 0 for low, 1 for medium, and 2 for high ROB, all yielding a total score ranging from 0 to 12 for each study. Low ROB is appointed to trials with total score from 0 to 4, medium ROB with total score from 5 to 8, and high ROB with total score from 9 to 12.

### 2.5. Statistical Analysis

All of the data was calculated by STATA 12.0 software package and Review Manager 5.3 software. The risk ratio (RR) with 95% CI was applied to analyze the dichotomous data. *χ* ^2^ and *I* ^2^ tests were used to assess statistical heterogeneity among included studies. If there was no heterogeneity across the trials, the pooled result was obtained by the fixed effect model; otherwise, the random effect model was used. Sensitivity analysis was conducted to estimate the stability of pooled result. Further, we employed Begg's funnel plot and Egger's test to test the publication bias.

## 3. Results

### 3.1. Search Result

**Figure 1:**
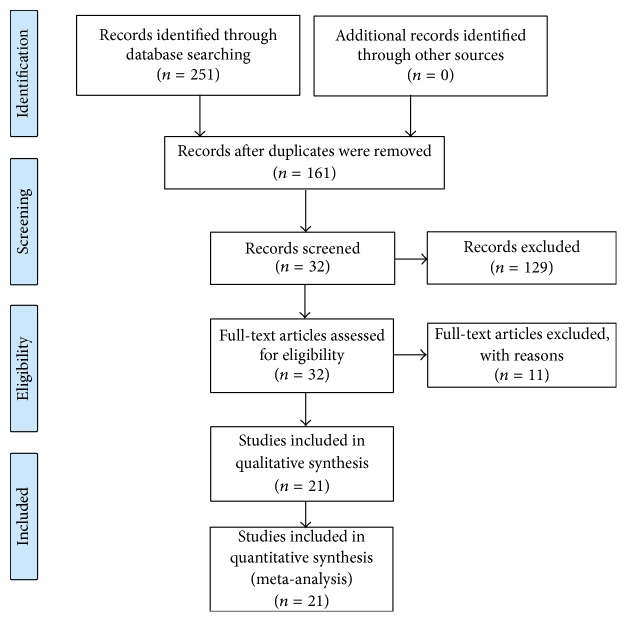
Flow chart of searching for included studies.

We conducted the systematic research on June 1_,_ and 251 potentially relevant references were yielded from online database. A total of 90 articles were excluded for duplication and 161 studies were entered next step. After further screening and eligibility assessment, 140 trials were excluded. Finally, 21 trials were selected as appropriate for inclusion in this meta-analysis. The flow chart showing the selection process was presented in Figure 1.

### 3.2. General Characteristic and Quality Evaluation

The selected trials [13–33] included 2234 advanced NSCLC patients, with 1122 and 1112 in the BJOEI group and control group, respectively, which were all RCTs and conducted in China. Patients' age varied from 18 to 79 years; in addition, the males outnumbered the females. All the included patients were in advanced stage. Nine [13–21] of the studies employed BJOEI plus GP regimen; four [22–25] employed the BJOEI plus TP regimen; four [26–29] employed BJOEI plus NP regimen; and four [30–33] employed BJOEI plus DP regimen. All of the studies reported the outcome of clinical efficacy; twelve [17–19, 21, 23–26, 29–31, 33] reported the outcome of quality of life; six [17, 19, 21–23, 25] showed the outcome of nausea and vomiting; eight [16, 18, 19, 21–25] provided the outcome of leukopenia; and four [19, 21, 22, 25] provided the outcome of thrombocytopenia. The general characteristics of included trials were shown in Table 1. For quality evaluation, all trials presented moderate ROB. The detailed ROB in different terms of each study was displayed in Table 2.

### 3.3. Objective Response Rate

**Figure 2:**
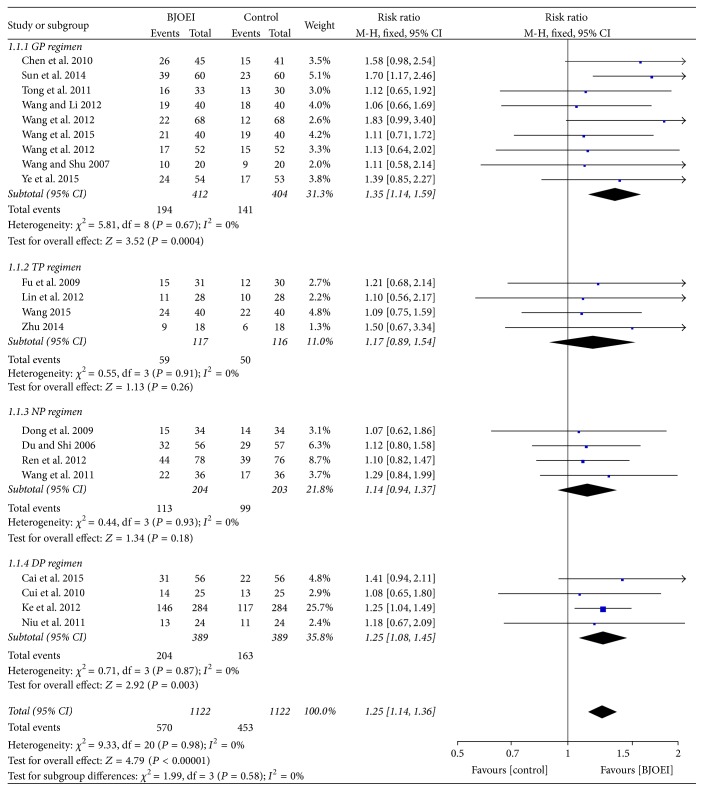
Meta-analysis of risk ratio for objective response rate of* Brucea javanica *oil emulsion injection combined with chemotherapy.

All included studies reported ORR in each arm. The heterogeneity analysis showed that no significant heterogeneity was found (*I* ^2^ = 0.00%; *P* = 0.98), and we applied the fixed effect model in the pooled analysis. As shown in Figure 2, the pooled result indicated a better RR in BJOEI treatment group than in control group (RR = 1.25; 95% CI: 1.14–1.36; *P* < 0.00001). The results in subgroup analysis of GP regimen (RR = 1.35; 95% CI: 1.14–1.59; *P* = 0.0004) and DP regimen (RR = 1.25; 95% CI: 1.08–1.45; *P* = 0.003) also demonstrated the favorable outcome. However, there were no significant differences with regard to ORR between BJOEI group and control group in subgroup analysis of both TP regimen and NP regimen. The integrated RR for ORR in TP regimen subgroup was 1.17 (95% CI: 0.89–1.54; *P* = 0.26) and the pooled RR for NP regimen group was 1.14 (95% CI: 0.94–1.37; *P* = 0.18). Briefly, this meta-analysis indicated BJOEI plus chemotherapy improved tumor response.

### 3.4. Improvement of QOL

**Figure 3:**
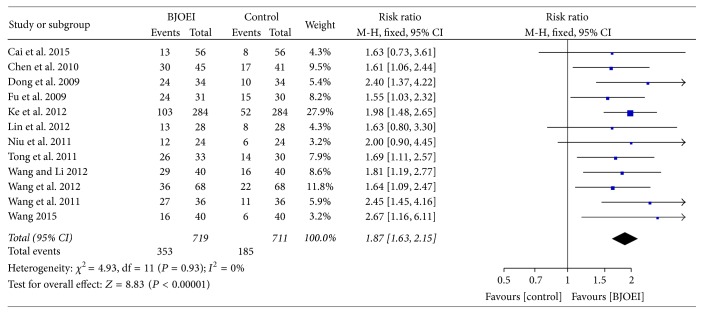
Meta-analysis of risk ratio for improvement of quality of life of* Brucea javanica *oil emulsion injection when combined with chemotherapy.

Twelve studies reported the improvement of KPS. No significant heterogeneity was found among these studies (*I* ^2^ = 0.00%; *P* = 0.93); thus we employed the fixed effect model in this meta-analysis. The pooled result demonstrated that BJOEI combined with chemotherapy could significantly improve the QOL (RR = 1.87; 95% CI: 1.63–2.15; *P* < 0.00001), which was illustrated in Figure 3.

### 3.5. Grade II or above Nausea and Vomiting

**Figure 4:**
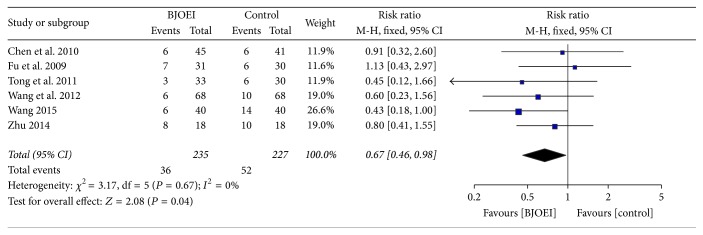
Meta-analysis of risk ratio for Grade II or above nausea and vomiting of* Brucea javanica *oil emulsion injection when combined with chemotherapy.

Six studies provided information about nausea and vomiting of the BJOEI treatment group and chemotherapy alone group. The heterogeneity test demonstrated no significant heterogeneity among the studies (*I* ^2^ = 0.00%; *P* = 0.67); thus we used the fixed effect model. As shown in Figure 4, the pooled results indicated that the BJOEI could decrease the risk of developing nausea and vomiting when patients received the chemotherapy combined with BJOEI (RR = 0.67; 95% CI: 0.46–0.98; *P* = 0.04).

### 3.6. Grade II or above Leukopenia

**Figure 5:**
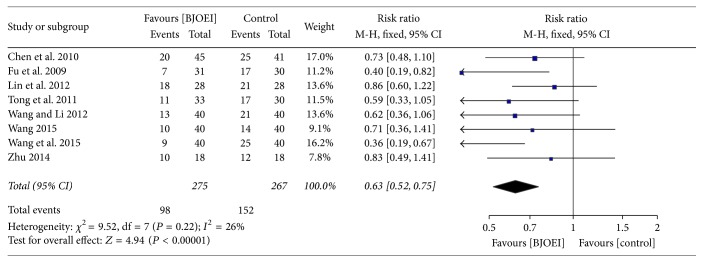
Meta-analysis of risk ratio for Grade II or above leukopenia of* Brucea javanica *oil emulsion injection when combined with chemotherapy.

There were eight studies that provided data on Grade II or above leukopenia. The heterogeneity among the included studies was not significant (*I* ^2^ = 26%; *P* = 0.22). As *I* ^2^ < 50%, they were considered to be homogeneous and a fixed effect model was employed for analysis (Figure 5). The result showed that the BJOEI combined with the chemotherapy decreased the incidence of Grade II or above leukopenia (RR = 0.63; 95% CI: 0.52–0.75; *P* < 0.00001).

### 3.7. Grade II or above Thrombocytopenia

**Figure 6:**
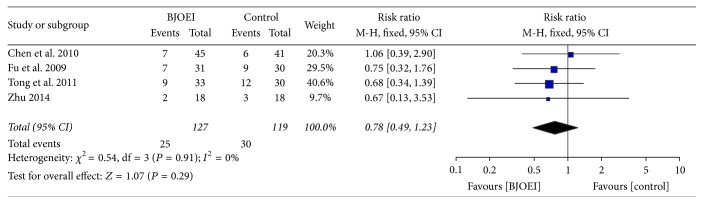
Meta-analysis of risk ratio for Grade II or above thrombocytopenia of* Brucea javanica *oil emulsion injection when combined with chemotherapy.

Four studies provided the data of Grade II or above thrombocytopenia in both arms with no statistical heterogeneity (*I* ^2^ = 0%; *P* = 0.91). Fixed effect model was employed in the meta-analysis. As illustrated in Figure 6, the pooled resulted showed that the BJOEI did not decrease the incidence of Grade II or above thrombocytopenia (RR = 0.78; 95% CI: 0.49–1.23; *P* = 0.29).

### 3.8. Publication Bias

**Figure 7:**
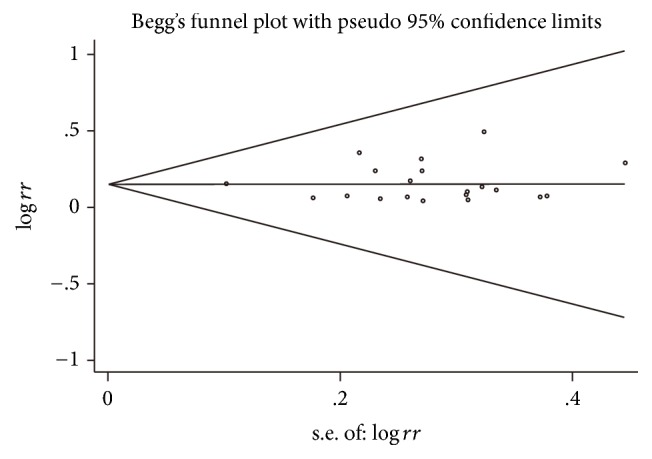
Publication bias of the included studies: funnel plot of objective response rate.

As all the eligible studies reported the outcome of tumor response, we chose to test the potential publication bias among the trials on ORR by Begg's funnel plot and Egger's test. As displayed in Figure 7, the funnel plot was symmetric, suggesting that no evidence of publication bias was found. Moreover, Egger's test provided evidence for no significant publication bias with *P* = 0.887.

### 3.9. Sensitivity Analysis

**Figure 8:**
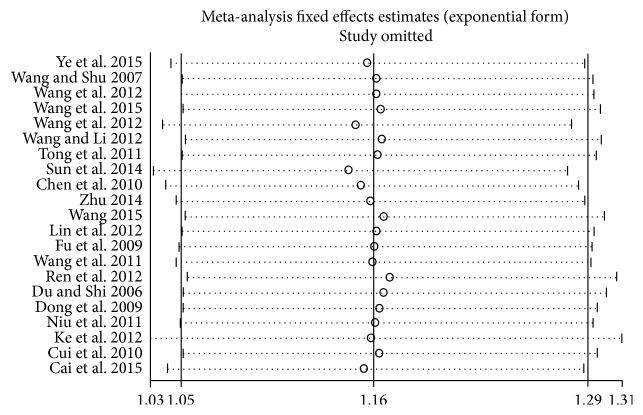
Sensitivity analysis of the included studies.

We performed a sensitivity analysis by sequentially omitting one single study to estimate the summary effect. We conducted the sensitivity analysis on the parameter of ORR for all the studied provided data on it. As shown in Figure 8, the combined effect after exclusion was close to that before exclusion, suggesting that the pooled analysis result was stable.

## 4. Discussion

As a powerful statistical analysis, meta-analysis can yield integrated result from individual study which focuses on the same issue [34]. We performed this meta-analysis to assess the effect of BJOEI plus chemotherapy on tumor response, quality of life, and side effects for advanced NSCLC patients. Twenty-one studies providing data on BJOEI plus chemotherapy versus chemotherapy alone were identified and analyzed comprehensively. As shown above, BJOEI combined with chemotherapy achieved better ORR (RR = 1.25; 95% CI: 1.14–1.36; *P* < 0.00001) and QOL (RR = 1.87; 95% CI: 1.63–2.15; *P* < 0.00001), and alleviated Grade II or above toxicity such as nausea and vomiting (RR = 0.67; 95% CI: 0.46–0.98; *P* = 0.04) and leukopenia (RR = 0.63; 95% CI: 0.52–0.75; *P* < 0.00001).

Apparently, baselines of the included studies were not consistent for the different chemotherapy regimens that the patients received. Therefore, we carried out a stratified analysis based on chemotherapy regimen for all the included studies on ORR. The results showed that when BJOEI was combined with TP regimen and NP regimen, no significant differences (*P* = 0.26 and *P* = 0.18, resp.) were found between the BJOEI group and control group. However, the pooled result (*P* < 0.00001) and the subgroup analysis of GP regimen (*P* = 0.0004) and DP regimen (*P* = 0.003) favored the BJOEI combined chemotherapy group. It seems that the sample sizes of TP regimen and NP regimen were too small to test validity. Besides, we evaluated the effect of chemotherapy regimen on quality of life but no significant difference was found, indicating that no matter which regimen the BJOEI was combined with, the addition of BJOEI during chemotherapy demonstrated a favorable outcome of QOL for advanced NSCLC patients. For publication bias, Begg's funnel plot and Egger's test were also applied in other parameters. The results showed that no publication bias was found for improvement of QOL (*P* = 0.92), nausea and vomiting (*P* = 0.72), leukopenia (*P* = 0.33), and thrombocytopenia (*P* = 0.94).

*Brucea javanica *oil (BJO) is the main ingredient in BJOEI. In vitro, BJO exhibited a potential ability to kill non-small-cell lung cancer cells [35]. The anticancer activity of BJO might be attributed to the following properties: inducing apoptosis [36], disturbing the cell cycle [36, 37], disrupting the cellular energy metabolism, and depressing the expression of vascular endothelial growth factor. Though the precise mechanism of this anticancer drug is poorly understood, our meta-analysis, together with the previous comprehensive analysis [38], corroborated the efficiency of BJOEI in clinical practice. In Wang's meta-analysis, twenty-two studies fulfilled the inclusion criteria and the pooled results showed that the addition of BJOEI during chemotherapy for NSCLC significantly increased the objective response rate (RR = 1.31; 95% CI: 1.18–1.45; *P* < 0.00001), improved the quality of life (RR = 1.78; 95% CI: 1.51–2.09; *P* < 0.00001), enhanced the immune function, and decreased the incidence of gastroenteric reaction (OR = 0.59; 95% CI: 0.44–0.80; *P* = 0.0007). Different criteria may lead to slightly different finding between these two meta-analyses, but the patients in BJOEI group did demonstrate superior objective tumor response, higher quality of life, and less side effects.

However, the ROB problem of included TCMs studies should be noticed. The quality assessment result indicated that no trial with low ROB was included, but all trails were with moderate ROB. Obviously, the relatively high risk of bias, resulting from faulty design of randomization, loss of double blinding, and allocation concealment, as well as the incertitude of patients' withdrawal, undermined the credibility of the synthesized results of our meta-analysis. McCulloch et al. [39] found that not accounting for ROB would have magnified the evidence of benefit and failed to detect nonsignificance of results. In their research, comparing with those with low ROB, studies with high ROB overestimated the efficacy of Chinese herbal medicines during fluorouracil-based chemotherapy for colorectal cancer by 16% improvement in tumor response (RR = 1.39, 95% CI: 1.18–1.63 versus RR = 1.20, 95% CI: 0.81–1.79), nearly 2-fold reduction of platelet toxicity (RR = 0.35, 95% CI: 0.15–0.84 versus RR = 0.65, 95% CI: 0.11–3.92), 2-fold reduction of vomiting toxicity (RR = 0.45, 95% CI: 0.33–0.61 versus RR = 0.87, 95% CI: 0.48–1.58), and 21% reduction in diarrhea toxicity (RR = 0.34, 95% CI: 0.20–0.58 versus RR = 0.43, 95% CI: 0.19–1.01). Although the quality of reporting RCTs of TCMs has been enhanced in the past decade, more and more academics realize that the percentage of high quality reports remains low, exacerbating the ROB problem relevant to TCMs trials [39–41]. Researchers should take responsibility for making registration of clinical trials [42], paying more attention to experiment design and methodological quality [43], and receiving education to write high quality report, so as to increase the credibility of TCMs studies.

Actually, limitations exist in our meta-analysis. First, we regarded EMBASE, PubMed, and the conference proceeding of ASCO as main sources of eligible studies, but all the included studies comparing BJOEI plus chemotherapy with chemotherapy alone for advanced NSCLC patients were searched in Chinese academic database. Second, double blinding and allocation concealment were not developed and implemented in all studies, incurring potential risks of selection bias and impairing the quality of this meta-analysis. Third, some of the studies which did not mention detailed characteristics of patients' age and gender distribution might also raise the risk of bias.

## 5. Conclusion

As one of the TCMs, BJOEI has been widely employed in China for many years. In recent year, the clinical practice indicated that the combination of BJOEI and chemotherapy not only improved the ORR and QOL, but also reduced the incidence of adverse events for the advanced NSCLC patients. Our meta-analysis did demonstrate and provide objective evidence to support the efficacy of BJOEI. Given that the quality of included studies is not high and there is a ROB problem in the meta-analysis, the anticancer effect of BJOEI should be further confirmed by higher-quality RCTs.

## Supplementary Material

The Preferred Reporting Items for Systematic Reviews and Meta-analysis (PRISMA) 2009 Checklist and information on the reasons why the articles are excluded after full-text reading, respectively.

## Figures and Tables

**Table 1:** Baseline characteristics of included studies.

Study ID	Age	Sex		N (B/C)	Intervention		Outcome	Criteria for outcome		KPS	Stage
		Male	Female		BJOEI group	Control group		Efficacy	Adverse event		
Wang and Shu 2007	27–72	22	18	20/20	GP + BJOEI 30 ml	GP	Efficacy rate	WHO criteria	—	>70	IIIB-IV
Chen et al. 2010	38–71	62	24	45/41	GP + BJOEI 30–40 ml	GP	Efficacy rate; quality of life; gastrointestinal reaction; myelosuppression	WHO criteria	WHO recommendations	>60	IIIB-IV
Tong et al. 2011	39–76	33	30	33/30	GP + BJOEI 30 ml	GP	Efficacy rate; quality of life; gastrointestinal reaction; myelosuppression	WHO criteria	WHO recommendations	≥60	IIIB-IV
Wang et al. 2012	52–74	94	42	68/68	GP + BJOEI 30 ml	GP	Efficacy rate; quality of life; gastrointestinal reaction	WHO criteria	WHO recommendations	>60	IV
Wang et al. 2012	32–76	77	27	52/52	GP + BJOEI 30 ml	GP	Efficacy rate	WHO criteria	—	≥60	III-IV
Wang and Li 2012	38–75	49	31	40/40	GP + BJOEI 30 ml	GP	Efficacy rate; quality of life; myelosuppression	RECST	—	≥60	IIIB-IV
Sun et al. 2014	40–70	74	46	60/60	GP + BJOEI 30 ml	GP	Efficacy rate	WHO criteria	—	>60	III-IVB
Wang et al. 2015	35–75	52	28	40/40	GP + BJOEI 20–30 ml	GP	Efficacy rate; myelosuppression	WHO criteria	WHO recommendations	≥60	III-IV
Ye et al. 2015	—	68	39	54/53	GP + BJOEI 30 ml	GP	Efficacy rate	RECST	—	>60	III-IV
Fu et al. 2009	29–71	40	21	31/30	TP + BJOEI 10 ml	TP	Efficacy rate; quality of life; gastrointestinal reaction; myelosuppression	WHO criteria	WHO recommendations	>60	IIIB-IV
Lin et al. 2012	30–70	41	15	28/28	TP + BJOEI 30 ml	TP	Efficacy rate; quality of life; myelosuppression	WHO criteria	WHO recommendations	≥70	IIIB-IV
Zhu 2014	40–70	25	11	18/18	TP + BJOEI 10 ml	TP	Efficacy rate; gastrointestinal reaction; myelosuppression	RECST	WHO recommendations	≥70	IIIB-IV
Wang 2015	35–75	58	22	40/40	TP + BJOEI 30 ml	TP	Efficacy rate; quality of life; gastrointestinal reaction; myelosuppression	WHO criteria	WHO recommendations	>70	IIIA-IV
Du and Shi 2006	27–72	76	37	57/56	NP + BJOEI 30 ml	NP	Efficacy rate; myelosuppression	WHO criteria	—	≥70	IIIB-IV
Wang et al. 2011	32–75	42	30	36/36	NP + BJOEI 40 ml	NP	Efficacy rate; quality of life	WHO criteria	—	>60	IIIB-IV
Dong et al. 2009	60–79	42	26	34/34	NP + BJOEI 30 ml	NP	Efficacy rate; quality of life; myelosuppression	WHO criteria	—	72–74	IIIA-IV
Ren et al. 2012	18–75	109	45	78/76	NP + BJOEI 40 ml	NP	Efficacy rate;	WHO criteria	—	>60	III-IV
Cui et al. 2010	—	32	18	25/25	DP + BJOEI 30 ml	DP	Efficacy rate	WHO criteria	—	≥60	IIIA-IV
Niu et al. 2011	≤75	37	11	24/24	DP + BJOEI 30 ml	DP	Efficacy rate; quality of life	RECST	—	≥60	III-IV
Ke et al. 2012	48–78	291	277	284/284	DP + BJOEI <70 ml	DP	Efficacy rate; quality of life; myelosuppression	WHO criteria	—	>60	IIIA-IIIB
Cai et al. 2015	42–78	64	48	56/56	DP + BJOEI 30 ml	DP	Efficacy rate; quality of life	WHO criteria	—	≥60	III-IV

BJOEI, *Brucea javanica* oil emulsion injection; GP, gemcitabine + platinum; TP, paclitaxel + platinum; NP, vinorelbine + platinum; DP, docetaxel + platinum; KPS, Karnofsky Performance Score.

**Table 2:** Risk of Bias scores for included studies.

Study ID	Random sequence generation	Allocation concealment	Blinding of participants and personnel	Incomplete outcome data	Selective reporting	Other bias	Total score
Moderate ROB							
Wang and Shu 2007	1	2	2	0	1	1	7
Chen et al. 2010	1	2	2	0	1	1	7
Tong et al. 2011	1	2	2	0	1	1	7
Wang et al. 2012	0	2	2	2	1	1	8
Wang et al. 2012	0	2	2	0	1	1	6
Wang and Li 2012	1	2	2	0	1	1	7
Sun et al. 2014	1	2	2	0	1	1	7
Wang et al. 2015	0	2	2	0	1	1	6
Ye et al. 2015	0	2	2	0	1	1	6
Fu et al. 2009	1	2	2	0	1	1	7
Lin et al. 2012	2	2	2	0	1	1	8
Zhu 2014	1	2	2	0	1	1	7
Wang 2015	1	2	2	0	1	1	7
Du and Shi 2006	2	2	2	0	1	1	8
Wang et al. 2011	1	2	2	0	1	1	7
Dong et al. 2009	1	2	2	0	1	1	7
Ren et al. 2012	1	2	2	0	1	1	7
Cui et al. 2010	1	2	2	0	1	1	7
Niu et al. 2011	0	2	2	0	1	1	6
Ke et al. 2012	1	2	2	0	1	1	7
Cai et al. 2015	2	2	2	0	1	1	8
